# Endoscopic Oncology

**DOI:** 10.1155/2014/785043

**Published:** 2014-03-31

**Authors:** Everson L. A. Artifon, Takao Itoi, Jose G. de la Mora Levy, Juan J. Vila

**Affiliations:** ^1^Surgery Department, University of Sao Paulo, Sao Paulo, Brazil; ^2^Gastroenterology Division, University of Tokyo, Tokyo, Japan; ^3^Gastroenterology Division, Cancer National Institute, Mexico City, Mexico; ^4^Gastroenterology and Hepatology Division, Complejo Hospitalario de Navarra, Pamplona, Spain

Endoscopy has evolved from a purely diagnostic tool to a highly selective minimally invasive therapy to treat both benign and malignant conditions. Thus, endoscopic treatment has become the mainstay of therapy for many oncological diseases in the gastrointestinal tract. The aim of this endoscopic treatment can be merely palliative as it is shown in the study by N. S. Ding et al. and in the study by Figueroa-Barojas et al. Ding et al. describe the outcomes of self-expandable metal stents (SEMS) deployment for managing 94 patients with malignant gastroduodenal outlet obstruction. The authors achieved an improvement in gastric outlet obstruction score in 90% of patients with a low rate of complications (5%) and a short hospital stay of only 3 days. On the other hand, Figueroa-Barojas et al. applied radiofrequency ablation to 25 unresectable malignant biliary strictures in 20 patients achieving a significant increase in the mean bile duct diameter of 3.5 mm at the expense of secondary postprocedural pain in 5 patients, one of them with development of pancreatitis.

But the endoscopic treatment for oncological diseases can be also applied with a curative aim. In the study by K. Ohata et al., the authors examine the efficacy and safety of endoscopic submucosal dissection (ESD) for treatment of 608 cases of colorectal neoplasms including adenomatous polyps and lesions with malignant superficial invasion. The authors divided patients into two groups: patients with lesions between 2 and 4.9 cm in size and patients with lesions bigger than 5 cm, showing an equal efficacy in resection of both groups (99.2% and 99% resp., *P* = 0.8), although complications were significantly more common with ESD for larger lesions (4.1% versus 9.9%, *P* = 0.03).

Endoscopy has also improved its diagnostic role in gastrointestinal diseases by means of supporting techniques such as chromoendoscopy with colorant staining or newly developed electronical advances such as narrowband imaging (NBI). In the study by E. Ide et al., the diagnostic yield of chromoendoscopy with Lugol's staining is compared with the diagnostic yield of NBI to detect high-grade dysplasia and intramucosal esophageal squamous cell carcinoma in 43 patients with achalasia. The authors found that both methods offer a similar sensitivity and negative predictive value.

Finally, in this special issue, a study by E. Balik et al. describing predictive parameters for failure in ERCP is included. In this study, previous hepatic biliary tract surgery, malignant infiltration of the ampulla, obstruction of gastroduodenal tract, and ulcerative duodenal disease significantly increased the failure rate.

*Everson  L. A.   Artifon* *Everson  L. A.   Artifon* *Takao  Itoi* *Takao  Itoi* *Jose  G.  de  la  Mora  Levy* *Jose  G.  de  la  Mora  Levy* *Juan  J.  Vila* *Juan  J.  Vila*

